# Using carbonized low-cost materials for removal of chemicals of environmental concern from water

**DOI:** 10.1007/s11356-018-1781-0

**Published:** 2018-03-26

**Authors:** Eva Weidemann, Mirva Niinipuu, Jerker Fick, Stina Jansson

**Affiliations:** 1 Department of Chemistry, Umeå University, SE-901 87 Umeå, Sweden; 2 Umeå Energi AB, SE-901 05 Umeå, Sweden; 3 Industrial Doctoral School, Umeå University, SE-901 87 Umeå, Sweden

**Keywords:** Hydrochar, Adsorption, Hydrothermal carbonization, Agro-industrial residues, Organic chemicals, Low-cost adsorbents

## Abstract

Adsorption on low-cost biochars would increase the affordability and availability of water treatment in, for example, developing countries. The aim of this study was to identify the precursor materials and hydrochar surface properties that yield efficient removal of compounds of environmental concern (CEC). We determined the adsorption kinetics of a mixture containing ten CECs (octhilinone, triclosan, trimethoprim, sulfamethoxasole, ciprofloxacin, diclofenac, paracetamol, diphenhydramine, fluconazole, and bisphenol A) to hydrochars prepared from agricultural waste (including tomato- and olive-press wastes, rice husks, and horse manure). The surface characteristics of the hydrochars were evaluated via diffuse reflectance infrared spectroscopy (DRIFTS), X-ray photoelectron spectroscopy (XPS), and N_2_-adsorption. Kinetic adsorption tests revealed that removal efficiencies varied substantially among different materials. Similarly, surface analysis revealed differences among the studied hydrochars and the degree of changes that the materials undergo during carbonization. According to the DRIFTS data, compared with the least efficient adsorbent materials, the most efficient hydrochars underwent more substantial changes during carbonization.

**Electronic supplementary material:**

The online version of this article (10.1007/s11356-018-1781-0) contains supplementary material, which is available to authorized users.

## Introduction

Water treatment poses a serious global challenge, in terms of providing safe sanitation and using limited water resources in an economical and efficient manner that ensures the availability of clean water for everyone. However, high costs and technical complexity limit the use of conventional water-treatment techniques, including sewage treatment, oxidation, filtration, and adsorption on activated carbon (Gupta and Suhas 2009; Nyenje et al. 2010). Additionally, even if current sewage treatment plants reduce the spread of bacteria and nutrients, some chemicals pass though the treatment plant unreduced (Snyder et al. 2003; Lindberg et al. 2014; Melvin and Leusch 2016). This is especially crucial for chemicals of environmental concern (CEC), e.g., antibiotics, pharmaceuticals, and biocides, that are designed to have biological effects. Studies have shown that some pharmaceuticals can be absorbed by crops, and subsequently enter both humans and livestock (Franklin et al. 2016). The availability of water treatment can be increased via simple, inexpensive techniques. For example, adsorption, which removes various contaminants even at low concentrations, is easily realized, but is costly due to the limited lifetime of adsorbents prepared from non-local or fossil feedstock (Gupta and Suhas 2009). By valorizing local low-cost feedstocks, such as agricultural or food industry residues, adsorption becomes affordable also in countries and regions with high water stress and limited economical resources (Mohan et al. 2014).

Biochars are carbon-rich porous materials obtained via the carbonization of feedstock materials, and they may be used as adsorbent materials due to their similarity to commercial activated carbons. Biochars generated from wet feedstocks are produced preferably through hydrothermal carbonization (HTC), an energy-efficient wet technique performed without drying of the raw materials before carbonization. The resulting biochar, or more specifically the hydrochar, is more hydrophobic than the raw material and is therefore easily dried (Escala et al. 2013; Vom Eyser et al. 2015). HTC was first described in 1913 (Bergius 1913) and nowadays is considered one of the most sustainable approaches for obtaining functional carbon-based materials (Hu et al. 2010; Titirici and Antonietti 2010). This process involves the use of organic materials (such as carbohydrates, manure, sludge, wood, food, and other agricultural wastes) as precursors (Hu et al. 2010; Berge et al. 2011; Oliveira et al. 2013; Falco et al. 2013). Hydrochars often retain much of the surface functionalities from the raw materials (mostly oxygen and hydrogen-containing acidic groups, e.g., phenolic, lactonic, carboxyl, and carbonyl groups (Wiedner et al. 2013)) which increase interactions with ionic or polar compounds (Liu et al. 2010). Although different types of biochars have gained attention recently and many potential feedstocks have been identified (Mohan et al. 2014), the data on these materials is inconsistent. This inconsistency results from the different carbonization methods and parameters as well as adsorbates and adsorption test parameters employed. Additionally, complex chemical mixtures and simulating real wastewater, should be investigated instead of focusing on solutions containing only one compound.

In this screening study, the CEC-removal efficiencies of four hydrochars prepared from low-cost feedstocks were determined. The sorption properties were elucidated through surface characterization where the role of surface area and surface functionalities in the removal of contaminants from water, was evaluated.

## Materials and methods

### Preparation of hydrochars

Four organic residues were used in this study: horse manure, skin and seeds from tomato, olive press residues, and rice husks. These materials are considered representatives of common agricultural residues in countries with high water stress.

The hydrochars were prepared in a stirred high-pressure lab-scale reactor with an internal volume of 1 L (Zhengzhou Keda Machinery and Instrument Equipment Co., Ltd., China). The walls of the reactor were heated by a detachable resistance heater. After each experiment, the heater was removed and the reaction was water-cooled. The four feedstock materials were covered with ultrapure water, resulting in mixtures with dry-matter content ranging from 11 to 24% (rice husks 11%, manure 12%, tomato waste 12%, and olive waste 24%) and subsequently carbonized at 220 °C for 2 h. Portions (~ 600 mL) of the wet sample were carbonized during each experiment and yields of 57–66% (66, 62, 57, and 59% for rice husks, manure, tomato waste, and olive waste, respectively) were realized. After carbonization, the chars were retrieved via filtration and dried overnight at 105 °C.

Prior to use, the chars were demineralized, i.e., dissolvable material was removed, and the surface functionalities were protonated by washing the chars in 0.1 M HCl until a clear water phase was obtained (Meng et al. 2013), rinsing with ultrapure water and filtering (Munktell, Qualitative filter paper grade 3/< 10 μm), and drying at 105 °C for 12 h. All chars were homogenized by grinding in a mortar, except for the tomato waste chars, which were blitzed for 4 × 2-s pulses at 10000 rpm (Grindomix GM 200, Retch). The dry chars were then stored in a desiccator.

### Preparation of model water

Our model water contained ten different CEC substances: two biocides: octhilinone (2-octyl-4-isothiazolin-3-one) and triclosan; three antibiotics: trimethoprim, sulfamethoxasole, and ciprofloxacin; four pharmaceuticals: diclofenac, paracetamol, diphenhydramine, and fluconazole; and the plastic additive bisphenol A in concentrations of 10 μg/L in ultrapure water. The corresponding trade names, structures, CAS (Chemical Abstracts Service) registry numbers, solubility in water, log *K*_OW_, and main applications are shown in Fig. S1 (Supplementary data). The tested substances were selected, owing to their widespread use in developing countries as well as their ability to pass unreduced through conventional sewage treatment plants and being emitted into the environment through effluent water (Snyder et al. 2003; Lindberg et al. 2014; Melvin and Leusch 2016).

### Batch adsorption experiments

The experiments were all performed at 20 °C and were conducted as follows: Triplicate adsorption tests were performed using 50 ± 2.5 mg hydrochar + 10 mL model water in 15 mL plastic tubes. After weighing the hydrochar, water was added to the tube, which was then agitated for 1, 3, 5, 8, 12, 18, or 25 min. Afterwards, ca 5 mL of water/hydrochar was removed from the tube using a syringe, passed through a 0.45-μm syringe filter (Filtopur, Sarstedt) into a glass vial, and weighed before addition of the internal standard. The internal standard consisted of isotopically labeled bisphenol A, triclosan, trimethoprim, sulfamethoxasole, ciprofloxacin, diclofenac, paracetamol, and fluconazole. Furthermore, the samples were all either analyzed directly or frozen until analysis, which occurred within 3 weeks of the experiment.

For each hydrochar, triplicate blank samples were prepared via agitation with ultrapure water. Leaching of the analytes by the tubes was investigated by agitating (for 25 min) triplicate tube blanks of 10 mL ultrapure water. Similarly, adsorption of the analytes to the tube walls was investigated through a triplicate tube adsorption test where 10 mL of the model water was agitated (for 25 min) in the tubes. In addition, the capacity of the chars (relative to that of commercially available active carbon) was assessed by agitating triplicate samples of general-purpose-grade powdered activated carbon (Fisher Scientific) with the model water for 25 min. A list of samples, agitation times, and blank samples is provided in Table S1 (Supplementary data).

### LC-MS analysis

The samples were all analyzed using a Thermo TSQ Quantum Ultra EMR (Thermo Fisher Scientific, San Jose, CA, USA) mass spectrometer coupled to a PAL HTC auto sampler (CTC Analytics AG, Zwingen, Switzerland). Two pumps (Surveyor and Accela, Thermo Fisher Scientific, San Jose, CA, USA) were used, and the separation was achieved with a Thermo Hypersil Gold AQ (50 × 2.1 mm, 5 μm) column. Analytes were ionized via heated electrospray (HESI) or atmospheric pressure photoionization (APPI) using a krypton lamp at 10.6 eV, in positive- or negative-ion mode. The settings, including the HESI/APPI ionization data, polarities, precursor/product ions, collision energies, tube lens values, quantification and qualification ions, and limits of quantification (LOQ), associated with the analysis are summarized in Table S2 (Supplementary data). The analytical system is based on column switching using six- and ten-port valves; the basic setup is described by Khan et al. (2012) (Khan et al. 2012). During analysis, a spectral resolution corresponding to a full width half maximum (FWHM) of 0.7 was used for both quadrupoles.

### Surface-area determination

The specific surface area of the hydrochars was determined via N_2_ adsorption and calculated using the Brunauer–Emmett–Teller (BET) theory. Degassing was performed at 120 °C.

### Diffuse reflectance infrared fourier transform spectroscopy (DRIFTS)

Approximately 10 mg of dry sample was manually ground with ca. 390 mg Fourier transform-infrared (FT-IR) spectroscopy-grade KBr (Merck, Darmstadt, Germany) in an agate mortar. The corresponding FT-IR spectra were recorded in diffuse-reflectance mode (Bruker IFS 66v/S; Bruker Optik GmbH, Ettlingen, Germany) under vacuum conditions, in accordance with the protocol described by Gorzsás and Sundberg 2014. Data in the spectral range of 400–5000 cm^−1^ was collected at a spectral resolution of 4 cm^−1^. In addition, pure KBr was used as the background, and the spectral range 400–3750 cm^−1^ was used in the subsequent multivariate analysis. Prior to multivariate analysis, the spectra were baseline-corrected (asymmetric least squares, lambda = 14,000,000, *p* = 0.01), smoothed (Savitzky-Golay filtering, polynomial order = 1, frame = 5), and total-area normalized, using the protocol described by Felten et al. 2015.

### X-ray photoelectron spectroscopy (XPS)

The XPS spectra were collected with a Kratos Axis Ultra DLD electron spectrometer using a monochromatic AlKα source operated at 120 W. An analyzer pass energy of 160 eV and a pass energy of 20 eV were used for acquiring wide spectra and individual photoelectron lines, respectively. The surface potential was stabilized by the spectrometer charge neutralization system. The binding energy (BE) scale was referenced to the C1s line (set at 285.0 eV) of aliphatic carbon, and the spectra were processed with the Kratos software. Powder samples for the analysis were gently hand-pressed into a pellet (directly on a sample holder) using a clean Ni spatula.

### Principal component analysis (PCA)

The differences between the materials were graphically visualized via Principal Component Analysis (PCA) (Jolliffe 2002). In PCA, the variation in a dataset is determined by extracting orthogonal principal components from a larger number of variables. The first principal component (PC1) accounts for the largest variation in the dataset, PC2 accounts for the second largest, and so on. Score plots were used to display distributions of the observations (here, the carbonized and non-carbonized materials) projected onto a plane. In these distributions, similar samples are closely grouped, whereas dissimilar samples are separated by large distances. The SIMCA-P software package (version 13.0, Umetrics AB, Sweden) was used for the PCA modeling and all variables were center-scaled. Furthermore, the number of significant components was determined via sevenfold full cross-validation (CV) (Stone 1973), and components with eigenvalues lower than two were removed from the model.

## Results and discussion

### Overall adsorption efficiency

**Fig. 1:**
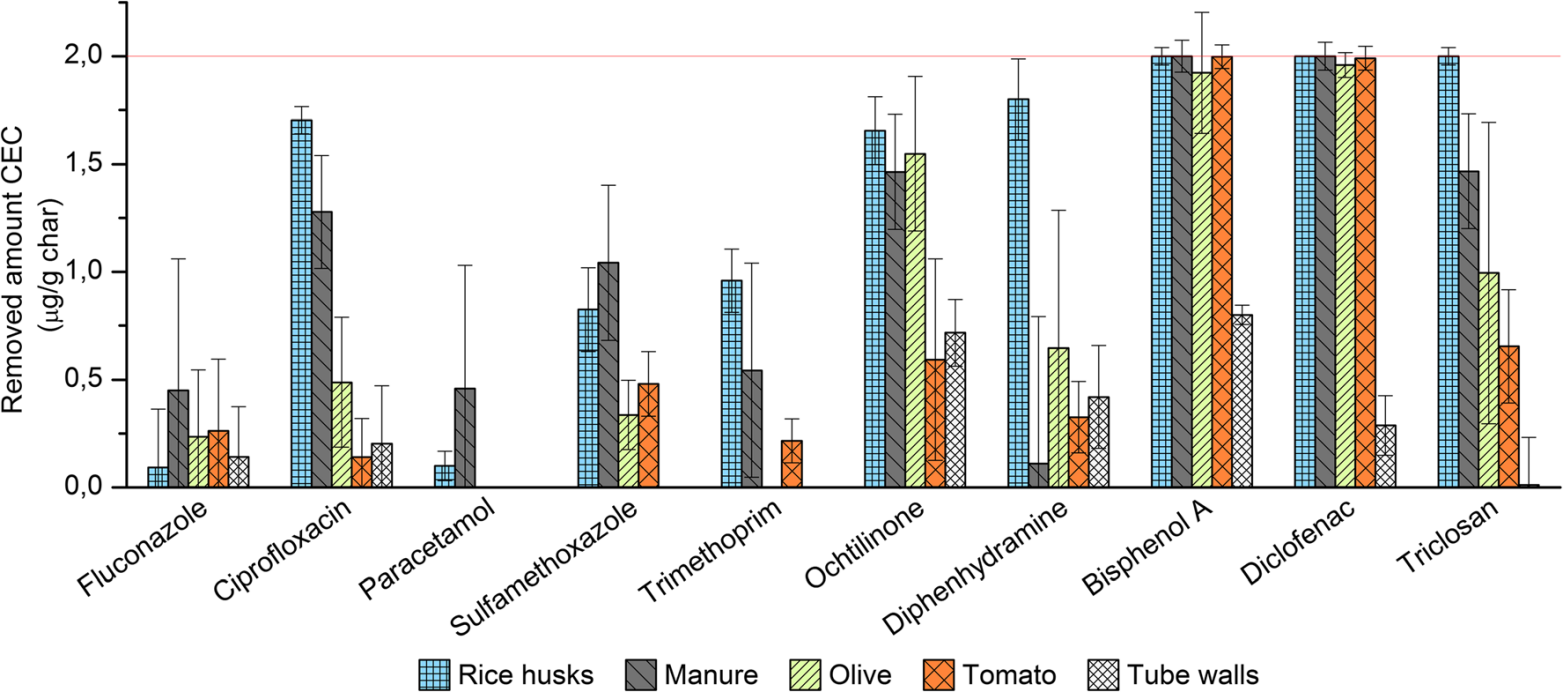
Total amount of removed CEC per gram of char. Removal was extremely rapid, thereby preventing the determination of gradients and, hence, the values displayed correspond to the average of all 21 samples. Error bars denote one standard deviation. Maximum amount removable: 2 μg/g hydrochar

The different hydrochars exhibited differing removal properties, and CEC removal from the water differed between substance and char. Furthermore, the substances were all rapidly removed, and the amount removed saturated after 1 to 3 min (see Figs. S2-S5 in Supplementary data for further details of the substances and chars). This rapid removal enabled the use of a total (overall)-removal efficiency for each substance and char, thereby improving the statistical accuracy of the removal results (Fig. 1).

Horse manure and rice husk chars exhibited the highest overall removal with CEC average removal efficiencies of 49 and 66%, respectively. These values are higher than the respective values (39 and 32%) associated with olive and tomato residue chars. Diclofenac and bisphenol A were nearly completely removed by all four chars. Similarly, rice husk char exhibited high efficiency (≥ 83%) of ciprofloxacin, diphenhydramine, octhilinone, and triclosan removal from the model water. Horse manure char was the most efficient material for removing paracetamol, fluconazole, and sulfamethoxazole, even though these compounds were generally removed to a low degree. Moreover, the CECs in the 18 blank samples were all lower than the LOQ (see Table S2, Supplementary data), and, after 25 min, the analytes were all removed by activated carbon. However, some of the analytes (especially diphenhydramine, octhilinone, and bisphenol A (Fig. 1)) were adsorbed onto the tube walls.

Similar results have been found in previous studies. Jung et al. (2013) reported nearly complete adsorption of bisphenol A using activated carbons from loblolly pine chips, while diclofenac was less efficiently removed and sulfamethoxazole the least adsorbed, and the adsorption followed the same order as in this study. Sulfamethoxazole removal by primary paper mill sludge char exceeded 50% in a study by Calisto and co-workers (2015), and in a study using activated sucrose hydrochar, the paracetamol removal was around 50% or less (Mestre et al. 2015). The low removal by these different hydrochars suggests that these types of compounds are not easily adsorbed from the solution. Nevertheless, comparisons should made with caution, because the concentrations in the above-mentioned studies were in the mg range. This is many orders of magnitude higher than the concentrations used in this study. Thus, it is likely that the solute concentration will affect the removal efficiency. This was indicated by Li et al. 2018, where ciprofloxacin removal efficiency was less for the lowest studied concentration compared to higher concentrations in the range of 150–500 mg/l.

**Fig. 2:**
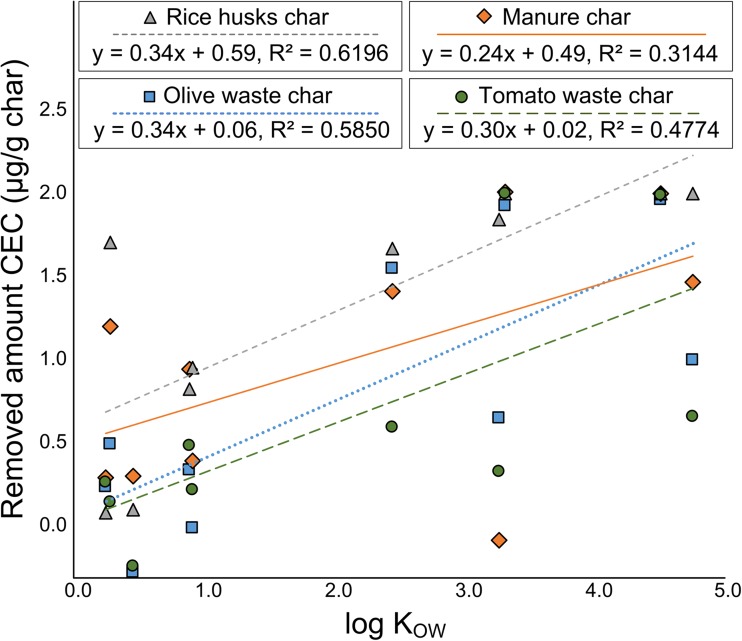
Correlation between the hydrophobicity of the adsorbate and the concentration adsorbed on each hydrochar

Correlations among the amount adsorbed, water solubility of each substance, and log *K*_OW_ were determined. No correlation was found between the removed amount of CEC and their solubility; however, in some cases, a positive correlation with log *K*_OW_ was observed (Fig. 2). Compared with their lower-log *K*_OW_ counterparts, the substances with higher log *K*_OW_ values were, in general, more efficiently removed by the chars. Removal by rice husk char exhibited the highest (*R*^2^ = 0.62, *p* = 0.0069) positive correlation with log *K*_OW_, suggesting that this removal has the best fitting linear correlation with log *K*_OW_ values of the CECs. The correlation obtained for carbonized olive waste and tomato waste, although lower than that of the carbonized rice husks, was still significant. Manure char exhibited the lowest overall correlation (*R*^2^ = 0.31, *p* = 0.09). When the amount removed was plotted as a function of log *K*_OW_, the steepest slopes, 0.34, 0.34, and 0.30, of the least square fit were obtained for the olive waste char, rice husk char, and tomato waste char, respectively. Compared with these plots, the plot corresponding to the horse manure char exhibited a more gradual slope (0.24), suggesting that the amount removed is more independent of log *K*_OW_. Low-log *K*_OW_ compounds, such as paracetamol and fluconazole, were most efficiently removed by horse manure char. This is attributed to the dissimilar surface properties that may promote different removal mechanisms. Hydrophobic molecules, in general, attach to surfaces via hydrophobic interactions, whereas their more hydrophilic counterparts are removed through other electrostatic interactions (e.g., hydrogen bonding) (Sun et al. 2012).

### Surface characterization

The surface properties of the carbonized material were investigated via three surface analysis techniques namely, BET, DRIFTS, and XPS. These analyses showed that the surface properties of rice husk and manure chars differ significantly from those of the tomato- and olive-residue chars.

In addition, BET-determined specific surface areas of 16.92 and 4.62m^2^/g were obtained for rice husk char and horse manure char, The surface area of the tomato and olive waste chars were below 1 m^2^/g (0.74 and 0.65 m^2^/g respectively), and these values were associated with high levels of uncertainty due to both the small surface area and the large fraction of volatiles on the char surfaces. This may have resulted from the relatively low carbonization temperature (220 °C). Moreover, considering that rice husk char had nearly fourfold higher surface area compared to horse manure char (16.92 vs. 4.62 m^2^/g), the overall removal for horse manure char (49%) was noteworthy. This indicates that the surface area constitutes only one factor that drives the adsorption.

**Fig. 3:**
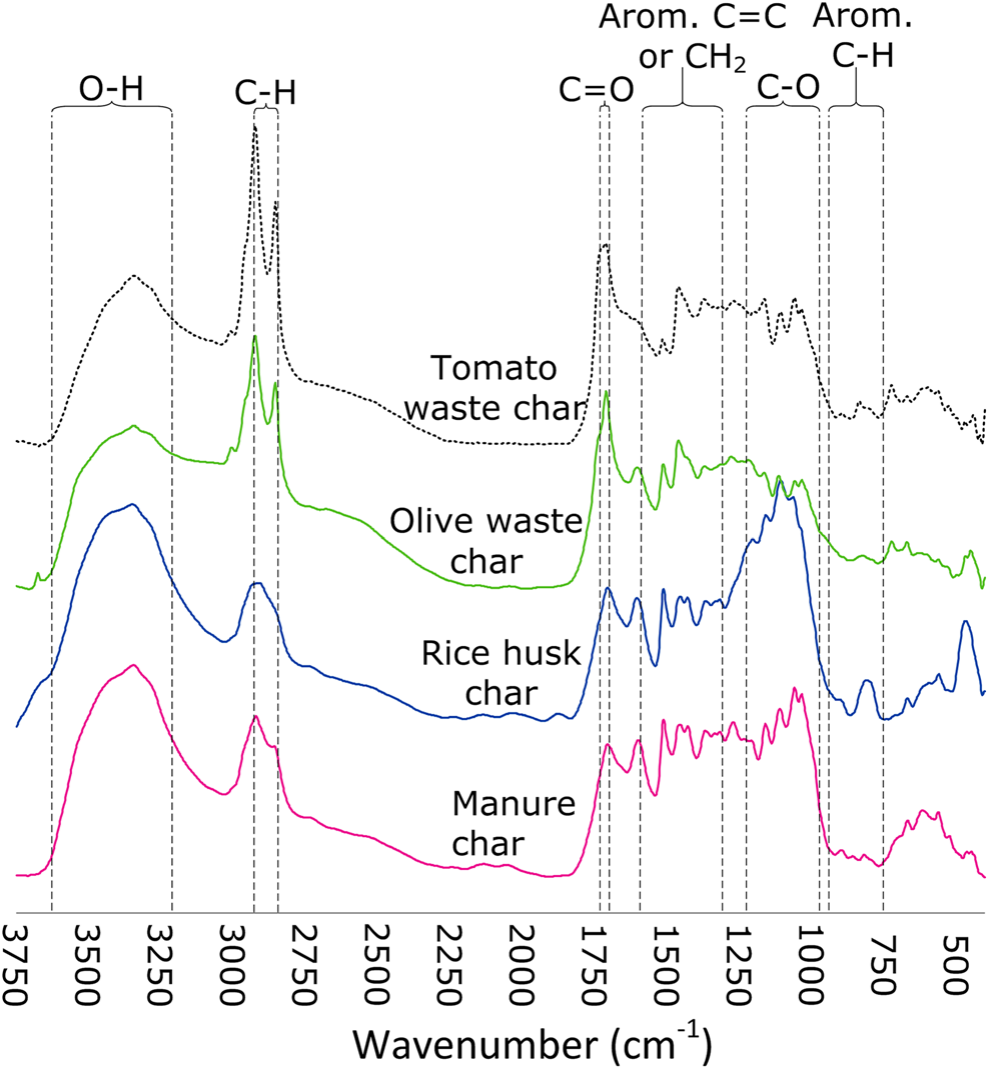
Baseline-corrected DRIFTS spectra of the treated materials

The DRIFTS spectra of the char materials and the raw materials are shown in Fig. 3 and Fig. S6 (Supplementary data), respectively. Broad O-H stretching bands from hydroxyl groups (Franca et al. 2010) occurred at 3500–3300 cm^−1^ for all samples, but occurred with higher intensity in the horse manure and rice husk chars. Furthermore, the materials all consist of aliphatic CH_2_, as indicated by the occurrence of C-H stretching vibrations at 2925 and 2853 cm^−1^, and small bands at 1450–1430 and 1370 cm^−1^ (Chen and Chen 2009). The bands associated with the olive and tomato waste were more intense than those associated with manure and rise husks before and after carbonization. Moreover, the sharp C = O stretching band occurring at 1750–1700 cm^−1^ results from carboxylic acids, esters, ketones, lactones, or aldehydes (Esteves et al. 2013). The band associated with the olive and tomato wastes is sharper than that corresponding to the manure and rice husks. Furthermore, the occurrence of bands at 1600–1450 cm^−1^ is indicative of aromatic ring C = C stretching in the samples (Chen and Chen 2009; Li et al. 2014), and small sharp bands at 900–700 cm^−1^ may have originated from aromatic C-H out-of-plane vibrations (Fang et al. 2014). Several narrow bands at 1300–1000 cm^−1^ are attributed to O-H bending and C-O stretching in ethers, alcohols, phenols, lactones or carboxyl acids, and anhydrides (Bustin and Guo 1999; Shuttleworth et al. 2015). The enhancement of these bands after hydrothermal carbonization of the materials is indicative of changes in the surface functionalities. Additionally, a large band at 1100–1000 cm^−1^ arising from the rice husk sample results probably from Si-O-Si in-plane vibrations, as suggested by XPS analysis results that revealed the presence of silicon (Table 1). The spectra of the treated materials are less diverse than those of the untreated materials, i.e., the intensities of C-H and C = O, and C-O bands vary more within untreated materials compared to the treated ones.

**Table 1:** Elemental composition (atomic-%) of the surface

Element	Rice husks	Horse manure	Olive waste	Tomato waste
C	54.30	74.52	87.84	83.71
O	37.69	23.57	11.51	14.71
N	–	1.52	0.64	1.58
Si	8.01	0.38	–	–

The elemental composition of the biochar surface is determined in further detail via XPS analysis (see Table 1). As the table shows, the carbon content of the olive and tomato waste chars is higher than that of the rice husk and horse manure chars. This result concurs with the DRIFT spectra, which exhibits high intensities for aliphatic carbon vibrations. In addition, silicon (bound as SiO_2_) occurs at a rather high concentration in the rice husk char, but constitutes only trace amounts of the horse manure char. Small amounts of carbon-bound nitrogen occur in all samples, except for the rice husk chars (Yang and Jiang 2014), and other elements occur at levels lower than the limit of detection (~ 0.1 atomic-%).

**Fig. 4:**
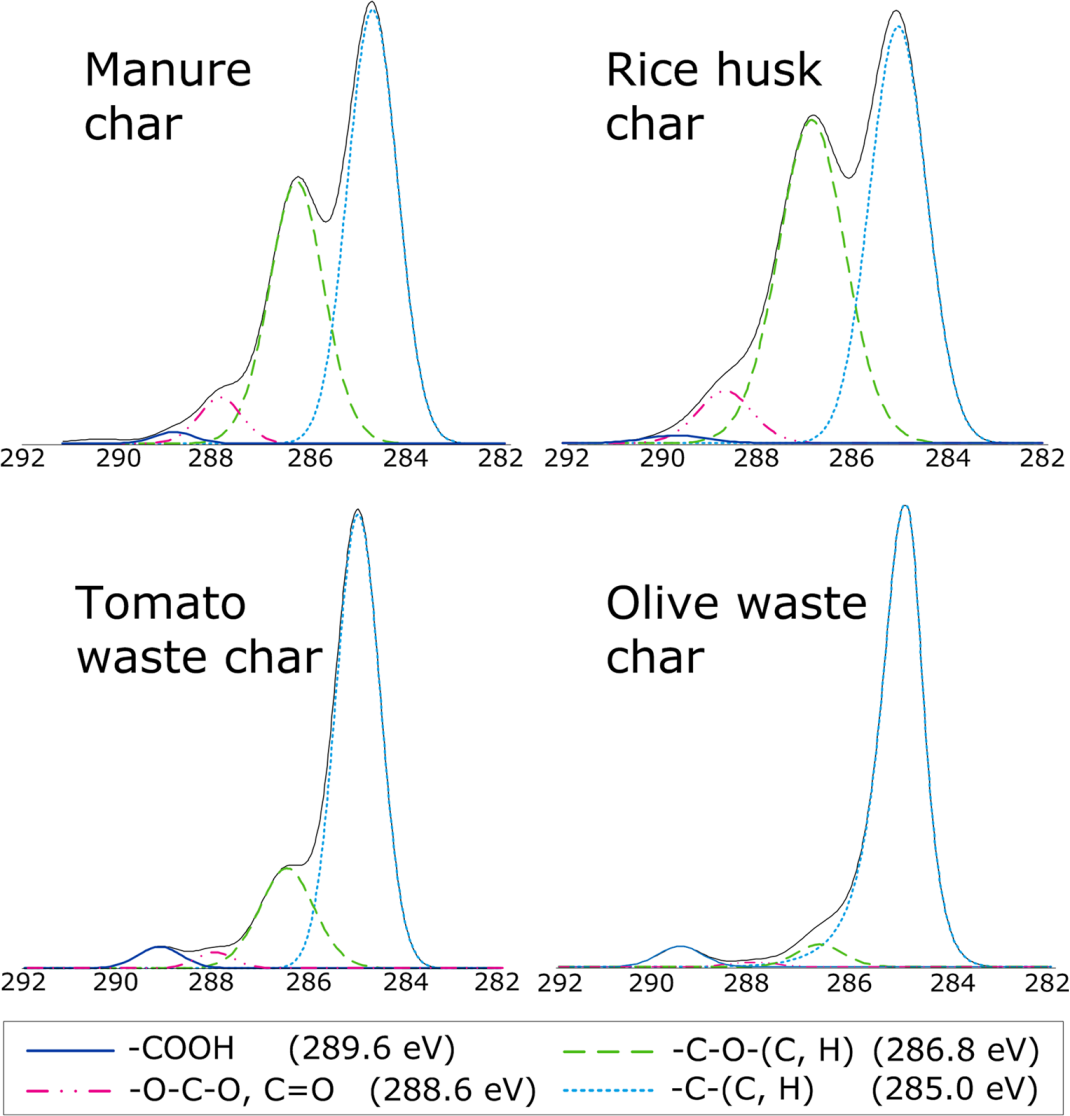
Deconvoluted C1s lines of the horse manure char, rice husk char, tomato waste char, and olive waste char

Figure 4 shows the deconvoluted C1s lines of the hydrochars. The peaks occurring at 284.9–285.0 eV correspond to aliphatic and/or graphitic carbon, the dominant species in all the chars. Compared with the olive and tomato waste chars, rice husk and manure chars contain higher fractions of carbon species with a single bond to oxygen, i.e., hydroxyls and ethers (at 286.5–286.8 eV), which support the previously presented DRIFTS data. Carbonyls at 288.1–288.6 eV and esters or carboxyl acids at 289.2–289.6 eV occurred in only small concentrations. Additionally, a minor line at 291.2 eV associated with the horse manure char, results from either a carbonate or π-π* shakeup satellite (Yu et al. 2012).

Surface analyses revealed substantial differences between the studied biochars. Although BET-determined surface areas varied between the best- (manure and rice husk chars) and worst-performing (tomato waste and olive waste chars) materials, this factor constituted only one of the several important features affecting this performance. DRIFTS and XPS revealed differences in the carbon content and O-functionalities, especially the OH content. Furthermore, the SiO_2_ and OH content of the rice husk and horse manure chars is higher than those of the tomato waste and olive waste chars, whereas the aliphatic carbon content is lower. Therefore, the rice husk and horse manure chars are more polar, and thereby may promote the removal of more polar molecules via H-bonding (Sun et al. 2012), than the other chars considered.

### Multivariate data modeling

**Fig. 5:**
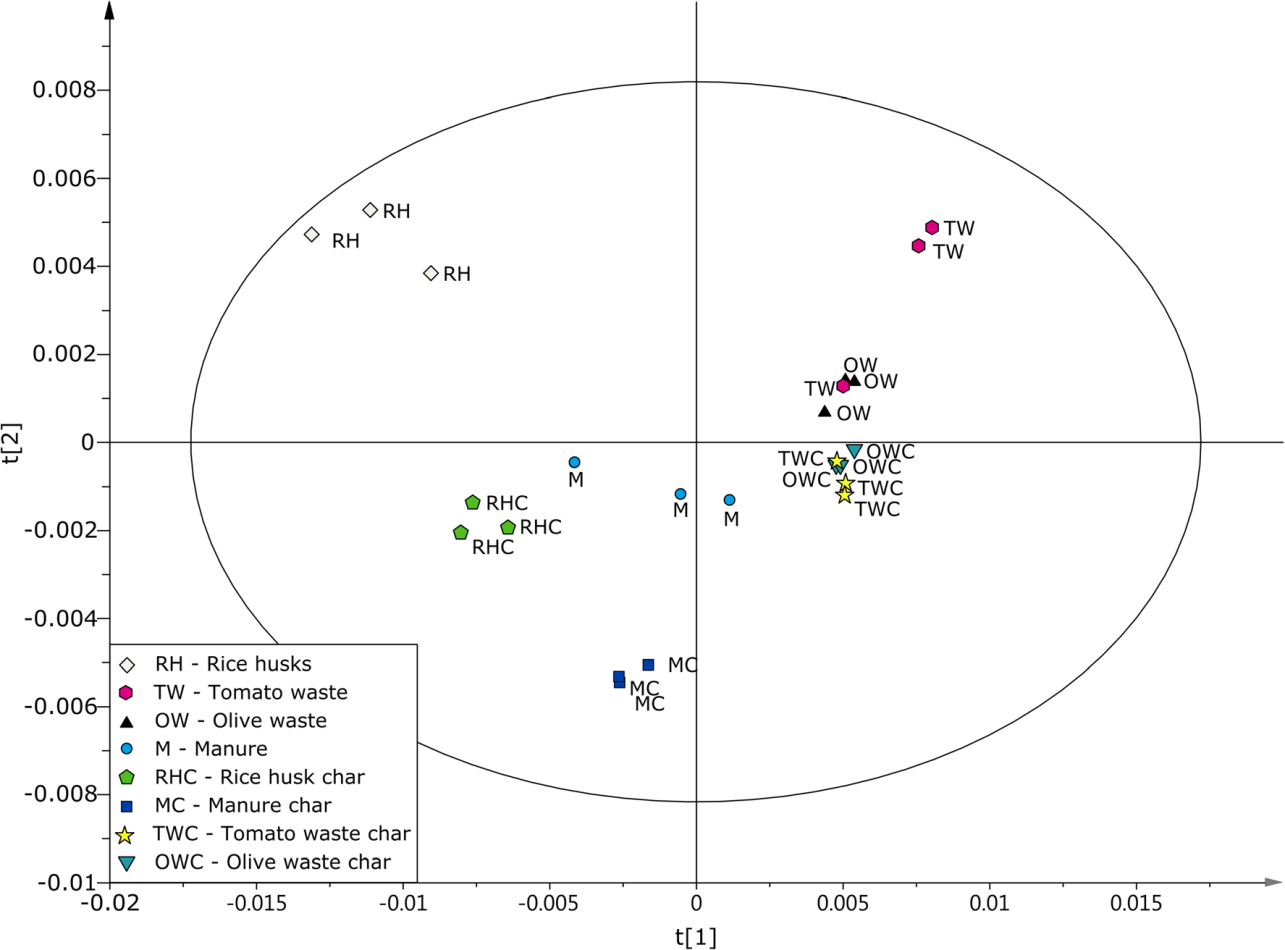
Plot showing the first two principal components of the PCA model of the DRIFTS spectra

The differences between the materials were elucidated by performing a PCA using the baseline-corrected DRIFTS spectra. The resulting model consists of four principal components that account for 86% of the variability (R2) with 69% predictive ability (Q2; see Fig. 5 for the score plot of the first two components). The PCA reveals that the treated materials are more homogeneous, as evidenced by the close grouping of the char replicates, than the untreated material. The PCA score plot also shows that the first principal component accounts for 57% of the variability (R2) and 42% of the predictive ability (Q2) (Fig. 5, t[1]). Furthermore, compared with that occurring for materials with poor removal capacity (i.e., tomato and olive waste), the variability in the DRIFTS spectra is higher for materials with good removal capacity (i.e., rice husks and manure). The inter-surface differences between high- and low-adsorption materials are larger than those between carbonized and non-carbonized materials (which are distinguished by the second principal component, t[2] with, variability (R2): 17%, predictive ability (Q2): 4%). The PCA model also revealed that the olive and tomato chars are very similar.

The overall results of the PCA model of the DRIFTS spectra may allow screening of untreated materials (based on surface functionalities) and prediction of removal capacities. However, a large database and multiple runs would be needed for highly heterogeneous untreated material (for example, manure or tomato waste).

## Conclusions

These findings demonstrate that carbonized low-value materials can remove CEC from water, but the removal efficiency varies with the feedstock. Furthermore, DRIFTS and XPS analyses revealed significant differences in the elemental composition and functionalities of the hydrochars. Multivariate analysis based on the DRIFTS data showed that, compared with those corresponding to the worst-performing hydrochars, larger differences between the treated and untreated materials occurred for the most promising hydrochars.

## Electronic supplementary material

ESM 1 (PDF 1085 kb)
