# Supplementary material for: Using carbonized low-cost materials for removal of chemicals of environmental concern from water
Source: Environ Sci Pollut Res Int. 2018 Mar 26;25(16):15793–801. doi: 10.1007/s11356-018-1781-0 (PMC5984643; doi:10.1007/s11356-018-1781-0)
Supplement: Supplementary file 1 — (PDF 1085 kb) [file 11356_2018_1781_MOESM1_ESM.pdf]

Supplementary Material

Using carbonized low-cost materials for removal of chemicals of environmental concern from water

Eva Weidemann, [†‡] *Mirva Niinipuu,* [†,§] *Jerker Fick,* [†] *and Stina Jansson* [†,] *

[†] Department of Chemistry, Umeå University, SE-90187, Umeå, Sweden

[‡] Umeå Energi AB, SE-90105, Umeå, Sweden

[§] Industrial Doctoral School, Umeå University, SE-90187, Umeå, Sweden

*corresponding author: stina.jansson@umu.se

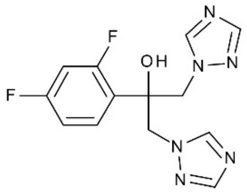

Fluconazole
CAS: 86386-73-4
$\log K_{OW}$: 0.25
Antifungal
Solubility: 1 mg/l (257°C)
WHO essential medicine (2015)

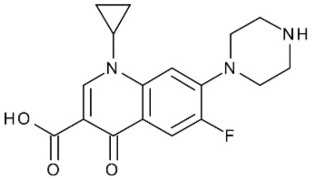

Ciprofloxacin
CAS: 85721-33-1
$\log K_{OW}$: 0.28
Antibiotic
Solubility: 30 000 mg/l (20°C)
WHO essential medicine (2015)

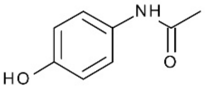

Paracetamol
CAS: 103-90-2
$\log K_{OW}$: 0.46
Analgesic and antipyretic
Solubility: 14 mg/l (25°C)
WHO essential medicine (2015)

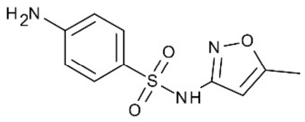

Sulfamethoxazole
CAS: 723-46-6
$\log K_{OW}$: 0.89
Antibiotic
Solubility: 610 mg/l (37°C)
WHO essential medicine (2015)

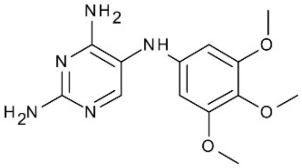

Trimethoprim
CAS: 738-70-5
$\log K_{OW}$: 0.91
Antibiotic
Solubility: 400 mg/l (25°C)
WHO essential medicine (2015)

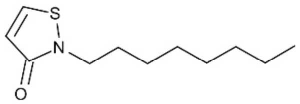

Ochtilinone
CAS: 26530-20-1
$\log K_{OW}$: 2.45
Anti-microbial fungicide
Solubility: 300 mg/l (n/a)

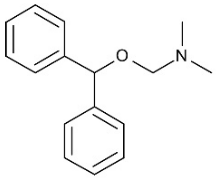

Diphenhydramine
CAS: 58-73-1
$\log K_{OW}$: 3.27
Antihistamine
Solubility: 3060 mg/l (37°C)

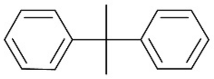

Bisphenol A
CAS: 80-05-7
$\log K_{OW}$: 3.32
Plastic additive
Solubility: 300 mg/l (25°C)

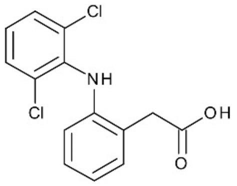

Diclofenac
CAS:15307-86-5
$\log K_{OW}$: 4.51
Nonsteroidal anti-inflammatory drug (NSAID)
Solubility: 2.37 mg/l (37°C)

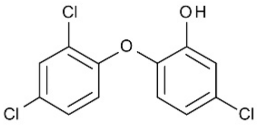

Triclosan
CAS: 3380-34-5
$\log K_{OW}$: 4.76
Antibacterial and antifungal
Solubility: 10 mg/l (20°C)

Figure S1. Trade name, structure, log k_{ow}-value, solubility in water (temperature), and the application for the molecules included in the model water. Solubility and $\log K_{OW}$ values were collected via PubChem database. The World Health Organization (WHO) essential medicines are

selected with due regard to disease prevalence and public health relevance, evidence of clinical efficacy and safety, and comparative costs and cost-effectiveness. (http://www.who.int/medicines/services/essmedicines_def/en/)

Table S1. Number of samples for each adsorption time and number of blank samples.

		Adsorption time (min)							
		1	3	5	8	12	18	25	Total no. samples
Samples	Horse manure	3	3	3	3	3	3	3	21
	Olive residue	3	3	3	3	3	3	3	21
	Rice husks	3	3	3	3	3	3	3	21
	Tomato	3	3	3	3	3	3	3	21
	Activated carbon	-	-	-	-	-	-	3	3
Blanks	Horse manure	-	-	-	-	-	-	3	3
	Olive residue	-	-	-	-	-	-	3	3
	Rice husks	-	-	-	-	-	-	3	3
	Tomato	-	-	-	-	-	-	3	3
	Tube	-	-	-	-	-	-	3	3
	Tube Adsorption	-	-	-	-	-	-	3	3

Table S2. Summary of ionization data, tube lens values, precursor/product ions, collision energies, and quantification and qualification ions, polarity and limit of Quantification (LOQ)

Name	Ion source [a]	RF [b] (V)	Precursor	Product	CE [c] (V)	Type [d]	Polarity	LOQ (ng/l)
Bisphenol A	APPI	56	228.0	213.0	13	Q	positive	50
		56	228.0	91.3	34	q	positive	
Bisphenol A IS	APPI	53	240.0	225.2	13	Q	positive	
		53	240.0	125.2	29	q	positive	
Ciprofloxacin	HESI	117	332.0	288.2	16	Q	positive	5
		117	332.0	231.1	35	q	positive	
Ciprofloxacin IS	HESI	106	336.0	318.0	20	Q	positive	
Diclofenac	HESI	83	295.9	214.1	33	Q	positive	5
		83	295.9	215.1	19	q	positive	
Diclofenac IS	HESI	76	300.0	219.1	19	Q	positive	
		76	300.0	254.1	13	q	positive	
Diphenhydramine	HESI	73	256.1	165.1	37	Q	positive	1
		73	256.1	167.1	13	q	positive	
Fluconazole	HESI	91	307.1	238.1	16	Q	positive	2.5
		91	307.1	220.1	17	q	positive	
Fluconazole IS	HESI	99	311.0	223.2	19	Q	positive	
		99	311.0	242.2	17	q	positive	
Octhilinone	HESI	51	214.0	84.2	39	Q	positive	25
		51	214.0	102.2	16	q	positive	
Paracetamol	HESI	81	152.0	65.4	32	Q	positive	10
		81	152.0	93.2	20	q	positive	
Paracetamol IS	HESI	62	155.0	93.2	22	Q	positive	
		62	155.0	111.2	15	q	positive	
Sulfamethoxazole	HESI	92	254.0	156.0	15	Q	positive	10
		92	254.0	108.2	22	q	positive	
Sulfamethoxazole IS	HESI	97	260.0	162.1	15	Q	positive	
		97	260.0	114.2	23	q	positive	
Triclosan	APPI	78	288.0	140.1	27	Q	positive	50
		78	288.0	218.1	22	q	positive	
Triclosan IS	APPI	51	300.0	230.1	23	Q	positive	
		51	300.0	234.0	35	q	positive	
Trimethoprim	APPI	106	291.0	123.2	25	Q	positive	1
		106	291.0	230.1	23	q	positive	
Trimethoprim IS	HESI	101	294.1	233.2	22	Q	positive	
		101	294.1	126.3	24	q	positive	

[a] APPI = atmospheric pressure photo ionization, HESI = heated electro spray ionization [b] RF voltage, [c] Collision energy, [d] Q = quantification ion, q = qualifying ion.

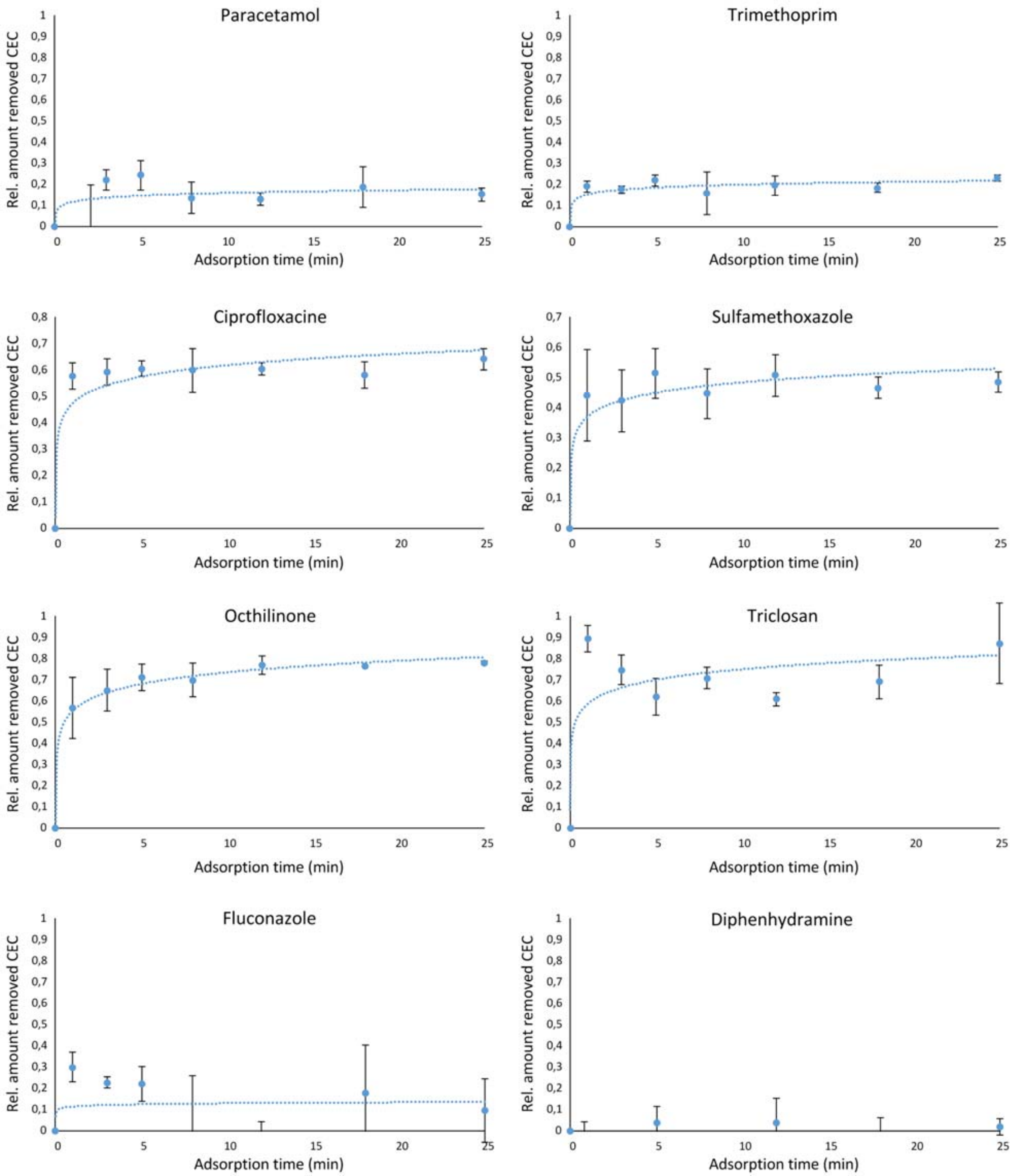

Figure S2. Adsorption kinetics for manure char. Bisphenol A and diclofenac (not shown) were entirely removed before 1 minute.

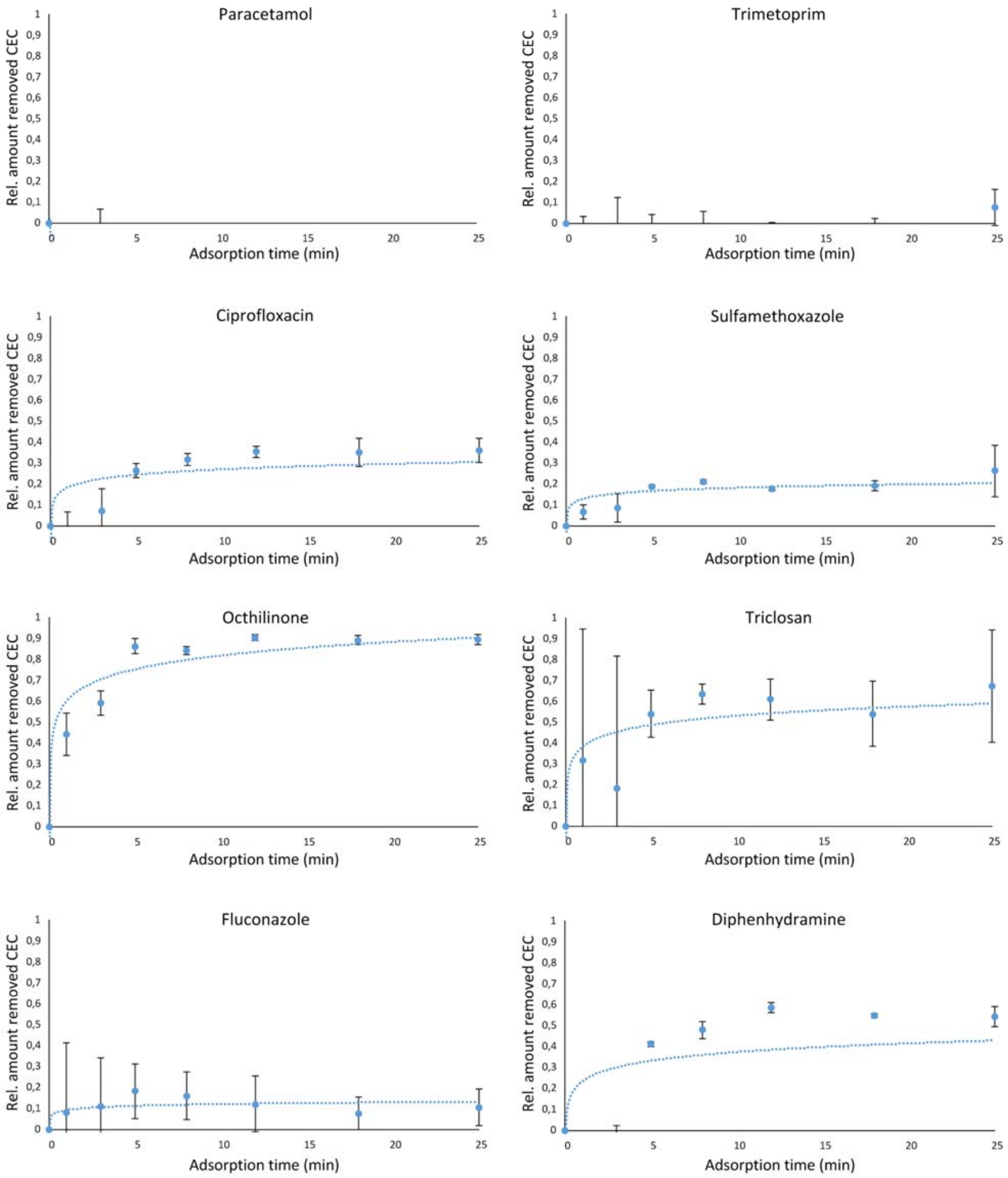

Figure S3. Adsorption kinetics for olive waste char.

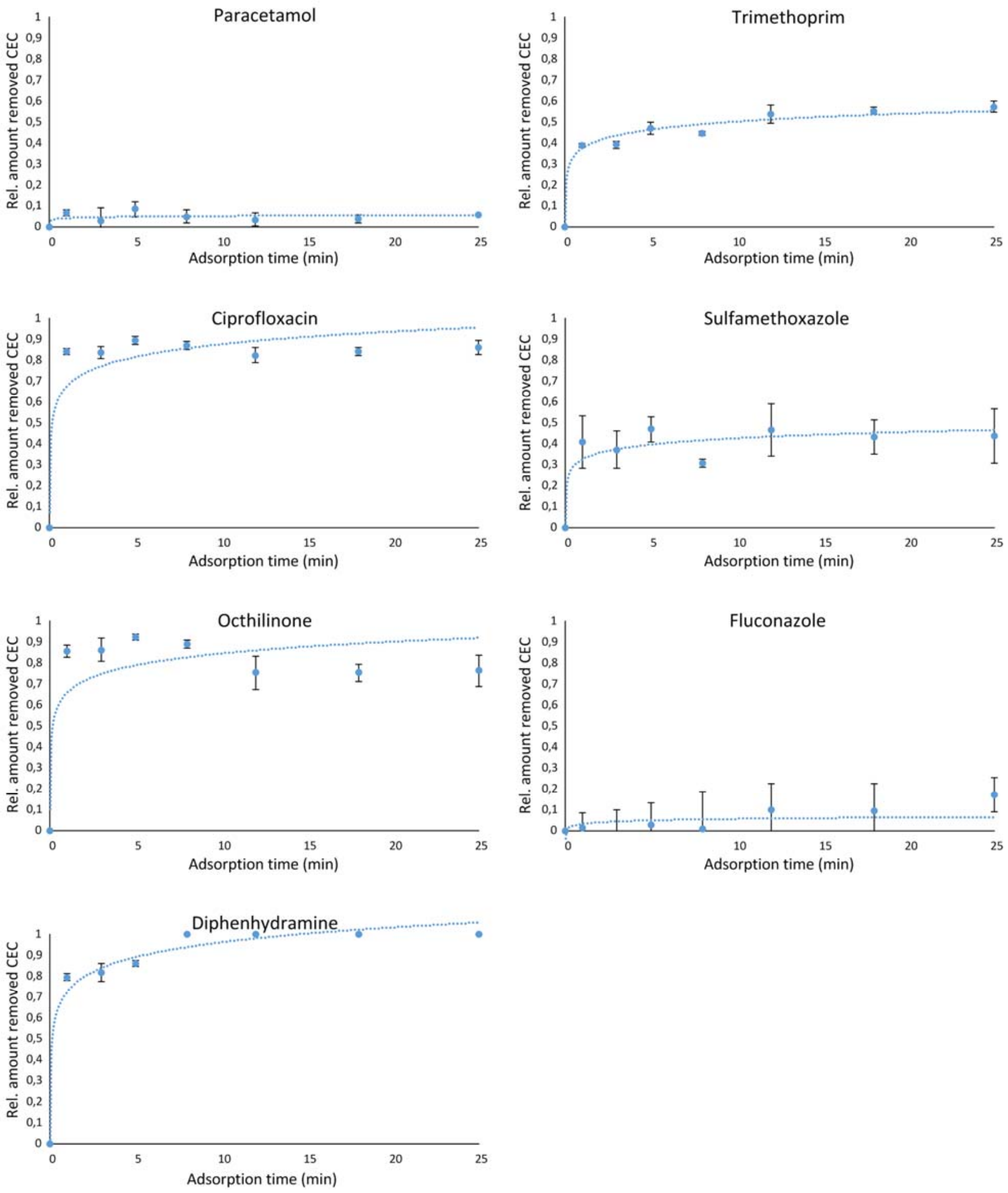

Figure S4. Adsorption kinetics for rice husks char.

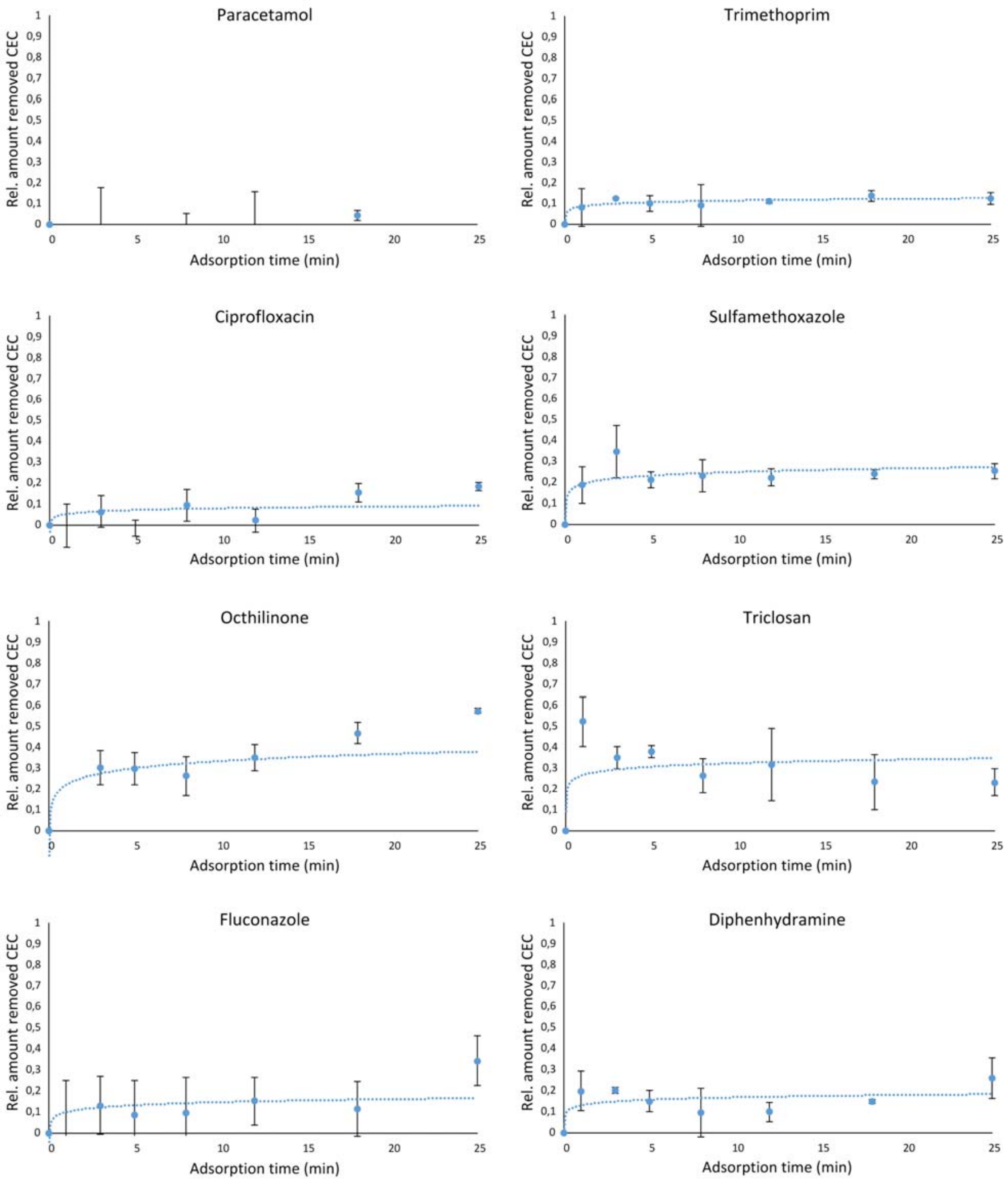

Figure S5. Adsorption kinetics for tomato waste char.

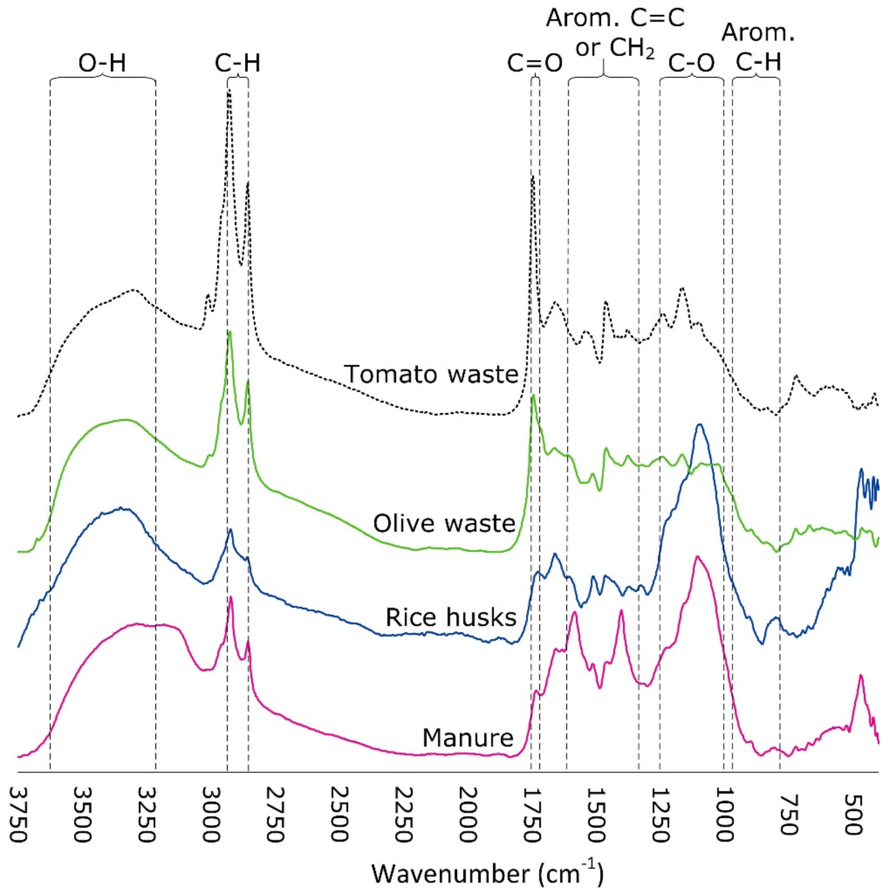

Figure S6. Baseline corrected DRIFTS spectra for the untreated materials.
